# The Efficacy of Transplanting Human Umbilical Cord Mesenchymal Stem Cell Sheets in the Treatment of Myocardial Infarction in Mice

**DOI:** 10.3390/biomedicines11082187

**Published:** 2023-08-03

**Authors:** Thang Quoc Bui, Nguyen Trong Binh, Truc Le-Buu Pham, Trinh Le Van, Nhung Hai Truong, Dang Phu-Hai Nguyen, Thao Thi-Thu Luu, Trang Nguyen-Xuan Pham, Tu Cam Tran, Huyen Thuong-Thi Nguyen, Nhu Thuy-Trinh, Phong Anh Tran

**Affiliations:** 1 Cho Ray Hospital, Ho Chi Minh City 700000, Vietnam; 2 Biotechnology Center of Ho Chi Minh City, Ho Chi Minh City 700000, Vietnam; plbtruc.snn@tphcm.gov.vn (T.L.-B.P.); nguyenphuhaidangbiotech@gmail.com (D.P.-H.N.); ntrang99710@gmail.com (T.N.-X.P.); 3 Faculty of Biotechnology, Ho Chi Minh City Open University, Ho Chi Minh City 700000, Vietnam; 4 Laboratory of Stem Cell Research and Application, University of Science, Ho Chi Minh City 700000, Vietnam; lvtrinh@hcmus.edu.vn (T.L.V.); thnhung@hcmus.edu.vn (N.H.T.); 5 Vietnam National University, Ho Chi Minh City 700000, Vietnam; cherrytsukuba2009@gmail.com; 6 Histology-Embryology-Pathology Department, Faculty of Medicine, University of Medicine and Pharmacy at Ho Chi Minh City, Ho Chi Minh City 700000, Vietnam; thaoluu@ump.edu.vn; 7 Institute of Tropical Biology, Ho Chi Minh City 700000, Vietnam; camtu79@gmail.com; 8 Divison of Human and Animal Physiology, HCMC University of Education, Ho Chi Minh City 700000, Vietnam; huyenntth@hcmue.edu.vn; 9 School of Biomedical Engineering, International University, Ho Chi Minh City 700000, Vietnam; 10 Interface Science and Materials Engineering Group, School of Mechanical, Medical and Process Engineering, Faculty of Engineering, Queensland University of Technology, Brisbane City, QLD 4000, Australia; phongbk@gmail.com

**Keywords:** myocardial infarction, mesenchymal stem cell, umbilical cord stem cell sheet, regenerative medicine

## Abstract

The transplantation of mesenchymal stem cell (MSC) sheets derived from human umbilical cords (hUCs) was investigated in this study as a potential application in treating myocardial infarction (MI). Two groups of hUC-MSC sheets were formed by populating LunaGel^TM^, which are 3D scaffolds of photo-crosslinkable gelatin-based hydrogel with two different cell densities. An MI model was created by ligating the left anterior descending coronary artery of healthy BALB/c mice. After two weeks, the cell sheets were applied directly to the MI area and the efficacy of the treatment was evaluated over the next two weeks by monitoring the mice’s weight, evaluating the left ventricle ejection fraction, and assessing the histology of the heart tissue at the end of the experiment. Higher cell density showed significantly greater efficiency in MI mice treatment in terms of weight gain and the recovery of ejection fraction. The heart tissue of the groups receiving cell sheets showed human-CD44-positive staining and reduced fibrosis and apoptosis. In conclusion, the hUC-MSC sheets ameliorated heart MI injury in mice and the efficacy of the cell sheets improved as the number of cells increased.

## 1. Introduction

Myocardial infarction (MI) is associated with a lack of blood supply to cardiac muscle cells, which results in cell death and necrosis. The average mortality rate of heart disease is 12.2%, and it causes 7 million deaths annually across the globe [1]. Stem cell transplantation is a potential therapy in regenerative medicine to treat various diseases, both acute and chronic diseases [2]. Mesenchymal stem cells (MSCs) have the potential to treat heart disease because the cytokines they secrete have been shown to regulate immune response, induce angiogenesis, and enhance tissue repair [3]. However, cell injection is rarely localized to the damaged tissue. For instance, it was discovered that just 1–2 percent of the bone marrow stem cells infused into the coronary artery were detected in infarct tissue [4,5]. Stem cell sheets, which are hydrogel layers loaded with stem cells, could be used to attach to infarcted tissue to deliver stem cells directly to the affected areas [6]. Stem cell sheet grafting has also been shown to increase cell survival in recipients [7,8].

In this study, we used a simple, yet effective method developed by our group, which used photo-crosslinkable gelatin-based hydrogel (LunaGel^TM^) as a 3D scaffold to encapsulate human umbilical cord-derived mesenchymal stem cells and form stem cell sheets. We investigated two different cell encapsulation densities and the effects of grafting the cell sheets to the injured heart tissue in an MI mouse model. 

## 2. Materials and Methods

### 2.1. Cell Sheet Preparation

As previously published [9], hUC-MSC suspension was mixed with LunaGel^TM^ (Gelomics, Kelvin Grove, Australia) solution in a 1 cm × 1 cm × 1 mm mold. Two cell concentrations of 10^5^ and 10^6^ cells per gel were chosen based on previous reports from other groups [10,11,12,13,14,15]. The cell–gel mixture was then cross-linked through irradiation with 405 nm light (LunaCrosslinker^TM^) following the manufacturer’s instructions. 

### 2.2. Animal

Twenty-five (25) healthy male BALB/c mice, aged 6 to 8 weeks, were acquired from the Laboratory of Animal Care and Use (Stem cell Institute, Ho Chi Minh City, Viet Nam). The animal experiments were approved by the Stem Cell Institute Animal Ethical Committee (No. 201201/SCI-ACE; Date: 25 December 2020) and were conducted in accordance with ARRIVE guidelines (Ref no. 201201/SCI-AEC).

### 2.3. MI Model

Endotracheal intubation and connection to the ventilator were performed after ketamine anesthesia was used on the mice. For ligation of the left anterior descending coronary artery with a 7–0 Prolene suture, a left lateral thoracotomy was made during mechanical ventilation. After that, the chest was closed, and the mice were then weaned from mechanical ventilation and extubated. After two weeks of surgery, the mice’s chests were re-opened with the same protocol with ventilator support to directly apply the cell sheets onto the MI area of the heart tissue.

### 2.4. Transplantation of the SCSs

The MI mice received one of the following treatments: (1) phosphate-buffered saline (PBS treatment), (2) LunaGel (LunaGel-only treatment), (3) a sheet of LunaGel with 10^5^ cells (“LunaGel + 10^5^ Cells” treatment) and a sheet of LunaGel with 10^6^ cells (“LunaGel + 10^6^ Cells” treatment). Treatment was applied directly onto the affected tissue areas. Additionally, five healthy mice were employed as a reference group. The effects of the cell sheets on the MI mice were studied for 2 weeks following transplantation.

### 2.5. Effects of the Stem Cell Sheets after Transplantation in MI

The body weight of the mice was measured daily. The left ventricle ejection fraction (EF) of the mice was evaluated with a SonoScape A5 ultrasound machine with the Linear 12 Mhz probe in M-mode following the manufacturer’s instructions; heart rate and blood pressure were measured using NIBP 76-0174 (Harvard, Holliston, MA, USA), and the mice movement activity index was calculated using UGO Basile 47,750 (Gemonio, VA, Italy).

### 2.6. Heart Histology

The mice were sacrificed at 4 weeks after surgery (i.e., 2 weeks after transplantation) as the end point of the study in order to harvest their heart tissue. The heart tissue was cryo-sectioned into 5 μm-thick slides after being soaked in OCT solution (Sigma, St. Louis, MO, USA). Slices of the heart were used for the following staining procedure.

### 2.7. Trichrome Staining

The collagen staining procedure was adapted from reference [16]. Briefly, the slices were preheated in Bouin solution at 60 °C for 15 min and then washed at room temperature (RT) under running water before being counterstained with hematoxylin (Sigma, St. Louis, MO, USA) for 5 min. The slices were first stained with fuchsin for one minute, washed, and then stained with phosphotungstic acid for five minutes and aniline blue for one minute, washed again, and finally incubated with acetic acid for five minutes. The dyed slices were dehydrated serially with ethanol and xylene before being imaged using a light microscope. The fibrosis percentage was calculated from the images using ImageJ/Fiji software version 2.9.0 (National Institutes of Health, Bethesda, MD, USA) [17].

### 2.8. Immunohistochemistry

The heart tissue slices were washed in buffered saline and blocked for an hour in blocking buffer (Tris buffer saline (TBS), with 1 percent bovine serum albumin (BSA, Sigma, St. Louis, MO, USA)). The slices that had been blocked were incubated with a primary anti-Annexin V antibody conjugated to FITC overnight 4 °C, or human CD44, followed by incubation with a secondary antibody conjugated with HRP for 1 h at RT. For the fluorescence conjugated antibody, the immunohistochemistry (IHC) slices were mounted using DAPI, and fluorescence images were captured using an IN CELL Analyzer 2500 (GE Healthcare, Chicago, IL, USA). According to the software’s instructions, the fluorescence image was quantified as the intensity. For HRP detection, the AEC kit was used as per the manufacturer’s instructions, and counterstaining with hematoxylin was carried out. Using a light microscope, the slices were examined at 2, 4, and 10× magnification.

### 2.9. The Transplanted Cell Sheets’ Cytokine Secretion

IHC staining with human CD44 was used to determine human cell survival, and the function of the transplanted cells’ secretion of VEGF (vascular endothelial growth factor), HGF (hepatocyte growth factor), Ang-1 (angiopoietin-1), eNOS (endothelial nitric oxide synthase), G-CSF (granulocyte colony-stimulating factor), GM-CSF (granulocyte-macrophage colony-stimulating factor, SDF-1 (stromal cell-derived factor 1), IGF-1 (insulin-like growth factor-1), and *HMGCR* (3-hydroxy-3-methylglutaryl-CoA reductase) was examined by real-time RT-PCR as below. 

### 2.10. Total RNA Extraction and cDNA Synthesis

Using a Monarch Total RNA Miniprep Kit (NEB, Ipswich, MA, USA), human stem cells and heart tissue from the mice’s transplanted SCSs were harvested to collect total RNA in accordance with the manufacturer’s instructions. Using a Maxima H Minus Reverse Transcriptase kit (Thermo Scientific, Waltham, MA, USA), 500 ng of extracted total RNA was reversed to cDNA. Thermo Scientific’s Maxima SYBR Green/ROX qPCR kit and a LightCycler^®^ 480 System (Roche Applied Science, Penzberg, Upper Bavaria, Germany) were used to further use the cDNA in a real-time RT-PCR experiment using the primers provided in Table 1. Using *GADPH* as a reference gene and healthy tissue as a normalization, the fold change of the genes was calculated using the Livak method [18].

### 2.11. Data Presentation

The results are presented as the mean ± standard deviation. The Student’s *t*-test and one-way ANOVA were used to carry out the statistical comparison between the groups. A *p*-value < 0.05 indicates a significant difference.

## 3. Results

### 3.1. Stem Cell Sheet (SCS) Transplantation Improved the MI Mice’s Appearance and Body Weight Gain

**Figure 1:**
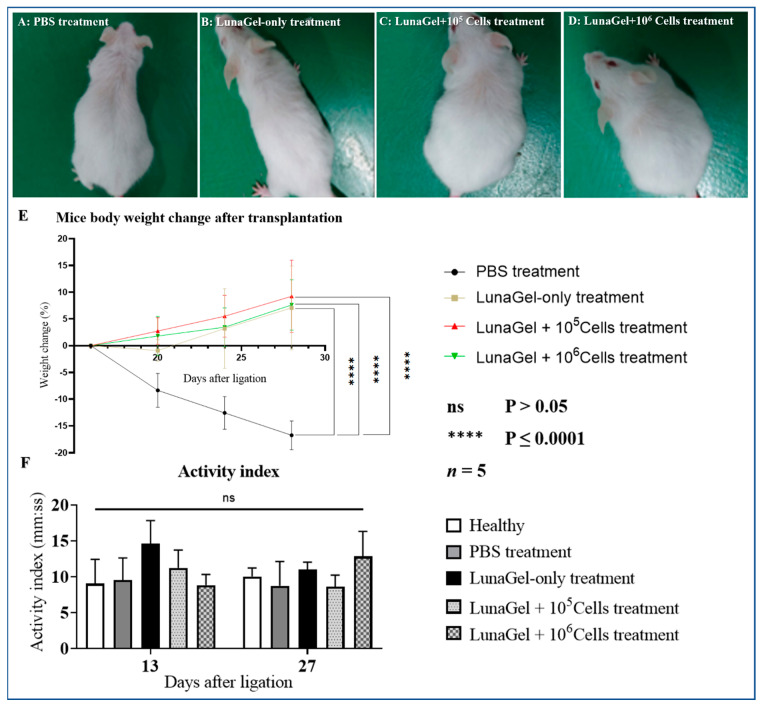
The appearance and weight results of the mice two weeks after treatment. (**A**–**D**) Apperance of mice after two weeks of receiving PBS treatment, LunaGel-only treatment, “LunaGel + 10^5^ Cells” treatment, and “LunaGel + 10^6^ Cells” treatment, respectively. (**E**) % weight change of mice after receiving the treatments. (**F**) The mice movement activity index before (day 13) and after (day 27) receiving the treatments.

The mice’s appearance after SCS transplantation is shown in Figure 1A–D. In general, compared to the groups receiving PBS and LunaGel-only treatment, the groups receiving stem cell sheets showed reduced hair loss and increased activity. Consistent with this appearance observation, the mice’s body weight gain was higher than the control mice (PBS treatment and LunaGel-only treatment). Moreover, there was no significant difference between the groups in the MI activity index as measured by the rotarod performance test (Figure 1F).

### 3.2. SCS Transplantation Improved Heart Function 

**Figure 2:**
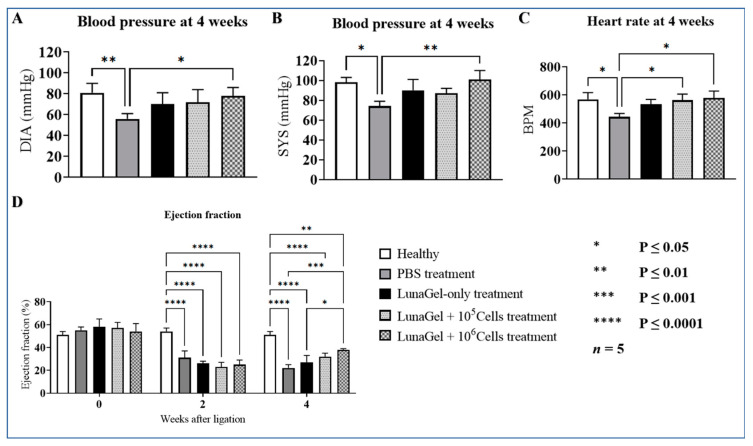
The effects of SCS transplantation on the heart function of the MI mice, including blood pressure (**A**,**B**), heart rate (**C**), and ejection fraction (**D**).

Next, we looked at the mice’s blood pressure, heart rate, and left ventricle ejection fraction to assess the effects of SCS transplantation on heart function in mice with MI. When compared to healthy mice, the blood pressure of the MI mice was significantly lower. In particular, the blood pressure of the MI mice was 74 ± 5 mmHg in SYS and 56 ± 5 mmHg in DIA, and the blood pressure of the healthy mice was averaged at 98 ± 5 mmHg in SYS and 81 ± 9 mmHg in DIA (mmHg, Figure 2A,B). The MI mice grafted with the higher number of cells showed higher pressure recovery, with readings of 101 ± 9 mmHg in SYS and 78 ± 8 mmHg in DIA. 

The heart rate of the MI mice was 444 ± 24 bpm, which is significantly lower than that of the healthy mice at 569 ± 48 bpm. The transplantation of LunaGel + cells increased the MI mice’s heart rate upon the study’s conclusion, and the groups treated with higher cell number showed slightly higher recovery with an average heart rate of 579 ± 49 bpm. 

At 4 weeks, the EF of the MI mice treated with PBS decreased to 0.22 ± 0.03, which is consistent with the blood pressure and heart rate results described above (Figure 2D). The MI mice that had LunaGel + cells treatment had improved EF in comparison to the MI mice receiving PBS; in particular, the EF averaged 0.32 ± 0.03 for the mice receiving LunaGel + 10^5^ cells and 0.38 ± 0.01 for the mice receiving LunaGel + 10^6^ cells. It is noteworthy that the MI mice receiving LunaGel-only treatment also had some improvement in their heart function compared to the MI mice receiving PBS treatment. 

### 3.3. Histopathology of the Heart Tissue

**Figure 3:**
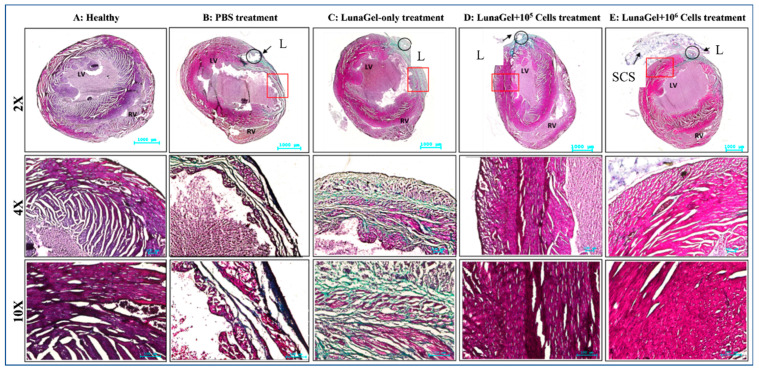
SCS transplantation in the MI mice histology. After 4 weeks of ligation, including 2 weeks of treatment, the heart tissues were collected and stained with trichrome. “L” indicates the ligated site of the coronary artery, “SCS” indicates the stem cell sheet, “LV” indicates the left ventricle, “RV” indicates the right ventricle, the red box indicates the magnification area.

Trichrome staining of the mice’s heart tissue revealed that the MI mice’s heart tissue had a higher percentage of fibrosis in the groups receiving PBS treatment or Lunagel-only treatment compared to the healthy mice (Figure 3).

The fibrosis region was reduced on the heart tissue of the MI mice that received SCS treatment (Figure 3). The grafted SCS was visible in the micrograph and appeared to bond with the heart infarcted tissue and serve as a protective membrane for the myocardium.

**Figure 4:**
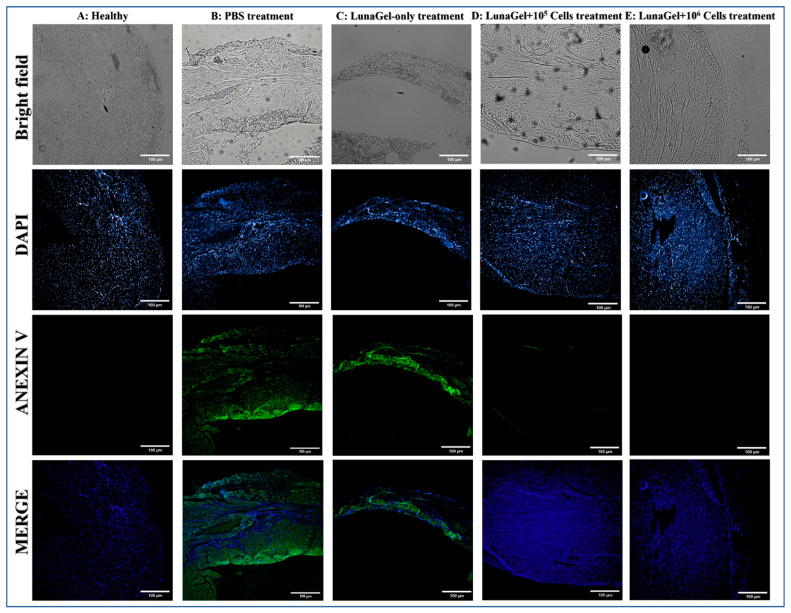
Immunohistochemistry of the heart tissue. After 4 weeks of ligation, including 2 weeks of treatment, the tissues were collected and stained with Annexin V (green) and DAPI (blue).

Figure 4 shows the results of cell apoptosis using Annexin V staining. In contrast with the normal muscle cells, which were negative for Annexin V, the MI mouse model promoted muscle cell death as seen in the groups treated with PBS (Figure 4A,B). Apoptosis significantly decreased in the groups grafted with LunaGel with or without cells. A thinner layer of muscle tissue positive for Annexin V was found in the LunaGel-only treatment group (Figure 4C), and the groups receiving LunaGel + cells treatment showed minimal positive staining (Figure 4D,E).

### 3.4. The Role of SCS Transplantation in Cytokine Secretion

**Figure 5:**
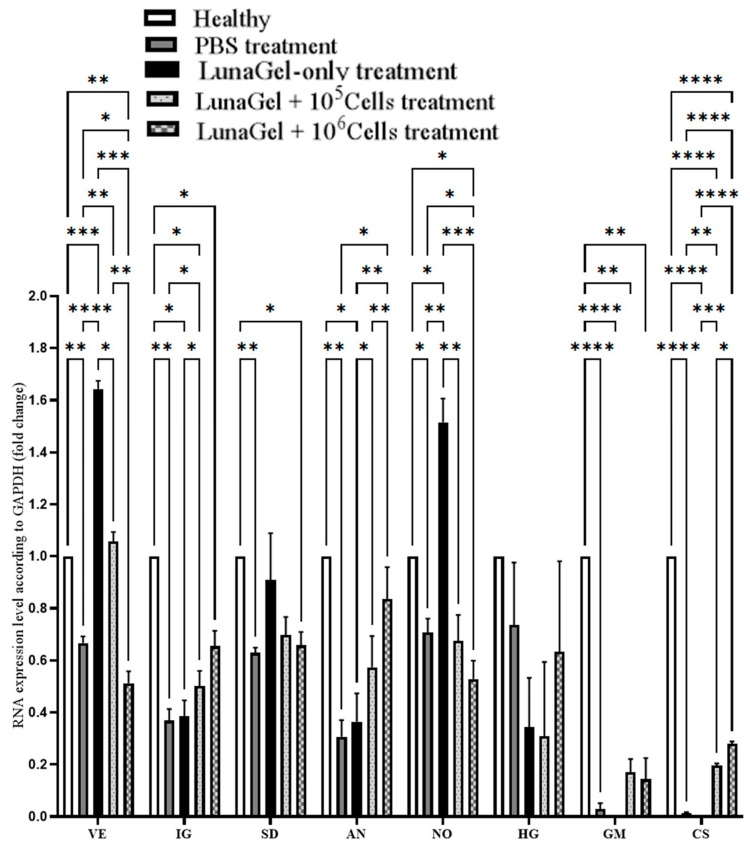
Gene expression associated with cytokine secretion. * *p* ≤ 0.05; ** *p* ≤ 0.01; *** *p* ≤ 0.001; **** *p* ≤ 0.0001.

Next, we assessed the effect of SCS transplantation on the cytokine secretion of angiogenesis-related genes, such as *Vegfa* and *Angpt1*, and cell survival factor-related genes, such as *Igf1*, *HMGCR*, *Nos3*, *hgf*, *csf2*, and *csf3*. The expression of these genes was computed as a fold change compared to the reference gene and normalized with the normal mice. Gene expression was frequently lower in the MI group compared to the normal mice group (Figure 5). Compared to the PBS control and LunaGel-only group, the SCS transplantation raised the expression of *Igf1*, *Angpt1*, *csf2*, and *csf3* but lowered the expression of the remaining genes in the MI mice. Thus, SCS transplantation increased angiogenesis and cell survival factor secretion in the MI mice. 

### 3.5. CD44 Immunohistochemistry of the Heart Tissue

**Figure 6:**
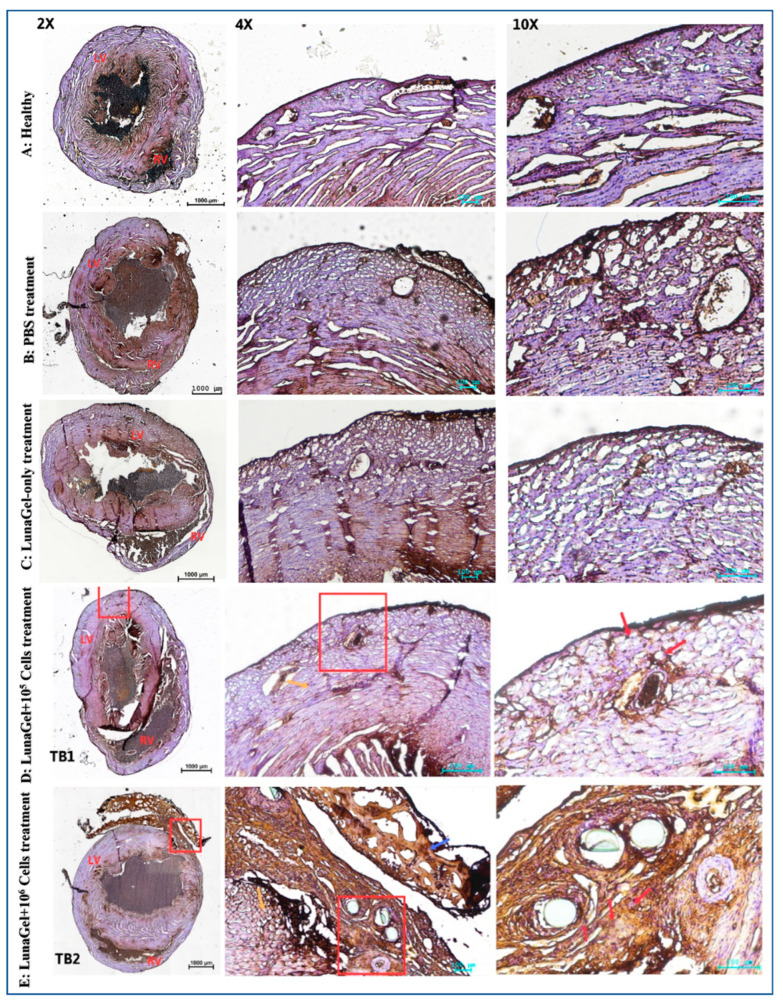
Antibody anti-human CD44 staining of the heart tissue. The red box indicates the magnification area, the blue arrow indicates the cell sheet, and the red arrow indicates the human CD44-positive cell.

Staining with the anti-human CD44 antibody showed positive staining in the grafts in the groups receiving the cell sheets (Figure 6). This result suggested that the SCS was capable of surviving after being grafted to the heart tissue for the 2-week duration of the experiment. 

## 4. Discussion

In this study, we showed that SCS transplantation in mice with MI improved cardiac function as measured by EF, heart rate, mice activity, and muscle cell regeneration. The higher number of cells in the sheets also showed greater improvement. In addition, the SCS sheets were found to be relatively stable until the end of the study, covering the injured tissue and promoting cell regeneration.

Evaluation of whole-heart fibrosis revealed that the SCS sheet reduced the percentage of fibrosis and improved tissue morphology (Figure 3). Upon conclusion of the study, the presence of human-CD44-positive cells (Figure 6) demonstrated that the graft was tolerated by the host immune system. The cell sheets were found to be attached to the injured tissue, and this was correlated with reduced tissue apoptosis and the improvement of damaged tissue compared to the control mice that received PBS as treatment (Figure 4). This improvement was also found to be higher in the group receiving higher cell numbers. This strongly suggests that LunaGel supported cell survival and prevented cell apoptosis.

The results of the gene expression of secreted cytokines indicated a mechanistic link between stem cell sheet grating and the improvement in heart function (Figure 5). In terms of angiogenesis cytokines, Angiopoietin-1 is a growth factor that promotes the formation of new blood vessels and the maturation of their morphology in in vitro [19] and in vivo studies [20]. In addition, ANG-1 plays an important role in anti-inflammation by repairing vessel lacerations at the site of damage [21]. Compared to the MI mice that received PBS as treatment, the gene expression of *Angpt1* was elevated in the MI mice with SCS transplantation in our study. The promotion of re-vascularization is expected to help restore blood supply to the infarct tissue. In addition, IGF-1 functions as a cell division, anti-apoptosis, and migration factor for smooth muscle cells [22]. Thus, IGF-1 induced the formation of new blood vessels [23], particularly in artery re-vascularization in vivo [24]. Similar to how SCS transplantation increased the expression of *Angpt1*, it also increased the expression of *IGF-1* in comparison to the control mice. 

In terms of cell survival and tissue regeneration, HGF is one of the most potent cell mitotic factors and a critical component of tissue wound repair [25]. HGF intervention has been shown to reduce tissue damage and improve cardiac function [26,27]. In our study, we showed that SCS transplantation induced HGF expression in MI tissue. HGF expression was also found to be higher in the group receiving higher cell numbers. 

## 5. Conclusions

We have demonstrated the application of hUC-MSC in the form of cell sheets formed by incorporating the cells into a photo-crosslinkable hydrogel (LunaGel^TM^) to treat myocardial infarction in mice. Two weeks after transplantation, significant improvement in heart function was observed. The improvement was found to be associated with the induced expression of cytokines related to anti-apoptosis and angiogenesis. Future studies should focus on understanding the role of the LunaGel in promoting cell viability after transplantation.

## Figures and Tables

**Table 1:** Primer sequences in the RT-PCR.

Gene Name		Sequence (5′-3′)	Gene ID
*Angpt1*	Forward	AATTTGTAAGCCGATCCGCC	GC08M108328
	Reverse	AGCCCCTTTCCTCTACCCTA	
*GADPH*	Forward	CAACTCCCACTCTTCCACCT	NM_008084
	Reverse	GAGTTGGGATAGGGCCTCTC	
*Csf3*	Forward	GGTTTAGCCCCGGAATTGAC	NC_000077.7
	Reverse	GGCTATAGTGACAGGTGGGG	
*Csf2*	Forward	TTTCACCAAACTCAAGGGCG	MGI:1339752
	Reverse	GTTCCTGGCTCATTACGCAG	
*Hgf*	Forward	CCTTGACTTAGCGATTGGGC	GC07M079863
	Reverse	CCCACATCATGCTTGCAGTT	
*Igf1*	Forward	GTCACACAAACTCACCACCC	GC12M101888
	Reverse	TTCTGATGTTGCACCCTCCT	
*Nos3*	Forward	GTCTTCCTCCCCTCCAGTTC	GC07P148937
	Reverse	AGCATATGAAGAGGGCAGCA	
*HMGCR*	Forward	GAGATCATGTGCTGCTTCGG	GC05P073440
	Reverse	CTTTGGGTTACGGGGTTTGG	
*Vegfa*	Forward	GCTGTAACGATGAAGCCCTG	NC_000083.7
	Reverse	CGCTCCAGGATTTAAACCGG	

## Data Availability

Not applicable.

## Abbreviations

Ang-1	Angiopoietin-1
ECM	Extracellular matrix
EF	Ejection fraction
eNOS	Endothelial nitric oxide synthase
G-CSF	Granulocyte colony-stimulating factor
GM-CSF	Granulocyte-macrophage colony-stimulating factor
HGF	Hepatocyte growth factor
HMGCR	3-hydroxy-3-methylglutaryl-CoA reductase
hUC-MSC	Human umbilical cord mesenchymal stem cell
IGF-1	Insulin-like growth factor-1 ()
LV	Left ventricle
MI	Myocardial infarction
MSC	Mesenchymal stem cell
RT	Room temperature
RV	Right ventricle
SCS	Stem cell sheet
SDF-1	Stromal cell-derived factor 1
VEGF	Vascular endothelial growth factor
